# Maternal Distress and Offspring Neurodevelopment: Challenges and Opportunities for Pre-clinical Research Models

**DOI:** 10.3389/fnhum.2021.635304

**Published:** 2021-02-12

**Authors:** Eamon Fitzgerald, Carine Parent, Michelle Z. L. Kee, Michael J. Meaney

**Affiliations:** ^1^Department of Psychiatry, Faculty of Medicine, Douglas Mental Health University Institute, McGill University, Montreal, QC, Canada; ^2^Ludmer Centre for Neuroinformatics and Mental Health, Douglas Research Centre, McGill University, Montreal, QC, Canada; ^3^Translational Neuroscience Programme, Singapore Institute for Clinical Sciences, Agency for Science, Technology and Research (A^*^STAR), Singapore, Singapore; ^4^Department of Paediatrics, Yong Loo Lin School of Medicine, National University of Singapore, Singapore, Singapore

**Keywords:** maternal distress, pregnancy, neurodevelopment, psychiatric disorders, pre-clinical models

## Abstract

Pre-natal exposure to acute maternal trauma or chronic maternal distress can confer increased risk for psychiatric disorders in later life. Acute maternal trauma is the result of unforeseen environmental or personal catastrophes, while chronic maternal distress is associated with anxiety or depression. Animal studies investigating the effects of pre-natal stress have largely used brief stress exposures during pregnancy to identify critical periods of fetal vulnerability, a paradigm which holds face validity to acute maternal trauma in humans. While understanding these effects is undoubtably important, the literature suggests maternal stress in humans is typically chronic and persistent from pre-conception through gestation. In this review, we provide evidence to this effect and suggest a realignment of current animal models to recapitulate this chronicity. We also consider candidate mediators, moderators and mechanisms of maternal distress, and suggest a wider breadth of research is needed, along with the incorporation of advanced -omics technologies, in order to understand the neurodevelopmental etiology of psychiatric risk.

The importance of the pre-natal period for neurodevelopment has been well-established through three major research streams. The first involves the follow-up of mothers and offspring subjected to an intense period of adversity during pregnancy. These studies include, amongst others, Hurricane Katrina, the North American Ice Storm of 1998 and mortality of a close relative (see below for references). The strength of these opportunistic, observational studies is the relative uncoupling of the exposure to the relevant event from the circumstances and conditions of the individual families and outcome measures, avoiding issues of reverse causality and supporting conclusions of cause – effect relations (e.g., it is unlikely that another's mental health caused Katrina!). The second stream includes longitudinal, epidemiological studies of mothers and offspring focusing on maternal symptoms of depression and anxiety, as well as measures of maternal stress. The strength of such studies is the prospective design and depth of measurement that permits detailed statistical modeling. These studies include mediational analyses that implicate candidate biological pathways. The third stream involves studies investigating the effects of stress imposed during pregnancy on developmental outcomes in the offspring of non-human species. The first two streams are descriptive studies of the associations between maternal conditions during pregnancy and neurodevelopmental outcomes in the offspring. The latter stream includes experimental studies that provide “biological plausibility” to support a causal relation between pre-natal maternal conditions and offspring development, as well as to identify candidate mechanisms. Collectively these studies form the basis for our understanding of the importance of pre-natal maternal health and well-being in offspring development.

## Etiology and Consequences of Adverse Maternal Mental Health in Pregnancy

There are two major streams of human epidemiological studies concerning maternal stress which provide very different models of maternal influence on neurodevelopment. The first refers to the sudden exposure to severe conditions such that they can be considered traumatic events. In such instances, pregnant women and their fetuses are exposed to severe adversity, the occurrence of which is independent of the pre-trauma maternal conditions. We appreciate the caveats to this assumption. While the nature and occurrence of the traumatic event may be random with respect to prevailing individual circumstances, the impact of the event is certainly moderated by pre-existing factors such as socio-economic status, social support networks, etc. However, these moderating influences would likewise vary across control populations such that the operative variable is that of the catastrophe. For the sake of this discussion, and with a plea for clemency, we will refer to these studies which examine sudden, catastrophic events as those of “maternal trauma” and those examining more sustained exposure to adverse environmental conditions, as well as symptoms of depression and anxiety, as “maternal distress” (borrowing the term from the original designations by Selye, 1975). We appreciate the clumsiness of this distinction. Trauma breeds lingering states of mental anguish (Yehuda and Meaney, 2018). Elsewhere, we (O'Donnell and Meaney, 2017) and others argue that symptoms of depression, anxiety and perceived stress *should not* be considered as synonymous with respect to either underlying mechanisms or effects on offspring development. Disingenuously, we now ignore this directive. The intent here is simply to note variation in the forms of maternal adversity and to distinguish those with a sudden onset at a specific perinatal period (i.e., trauma) from those that are chronic and, as argued below, generally operative across reproductive life (i.e., distress). We claim no other merit to this terminology. Unfortunately, studies with model systems of perinatal maternal “stress” commonly fail to recognize this important feature to the reality of maternal adversity in humans.

### Maternal Trauma

The study of maternal trauma derives largely from epidemiological surveys of the consequences of an environmental (i.e., Hurricane Katrina, Superstorm Sandy and the North American Ice Storm of 1998) or personal (i.e., the loss of a close relative) catastrophe. These are characterized by an acute onset of extreme trauma, which can be used to identify critical periods for psychiatric risk during gestation. For instance, in a large Danish population study of 1.38 million births, death of a close maternal relative, specifically during the first trimester, was associated with an increased incidence of schizophrenia in offspring (Khashan et al., 2008). Similarly, an increased risk of schizophrenia was seen in Dutch infants who were in their first trimester during the 1940 German invasion, with a male specific effect seen in the second trimester (Van Os and Selten, 1998). A pronounced effect on male fetuses was also seen following the 9/11 terror attacks, where across America there was an increased number of females born relative to males suggesting a selective loss of male fetuses (Bruckner et al., 2010). Similar effects were seen in the aftermath of the Chernobyl nuclear meltdown, but with a selective loss of males who were in their third month of gestation at the time of the incident (Peterka et al., 2004). Other environmental catastrophes such as the ice storm of 1998, superstorm Sandy and hurricane Katrina have been associated with reduced cognitive and language skills in offspring, as well as changes in infant temperament compared to pregnancies carried outside these periods (Laplante et al., 2008; Tees et al., 2010; Zhang et al., 2018).

Interestingly, trauma during the pre-conception period also confers an increased risk for adverse pregnancy outcomes. A large Swedish population study demonstrated that the loss of an immediate maternal relative 0–6 months *before conception* was associated with an increase in infant mortality during the first year of life, with no effect seen if bereavement occurred post-conception (Class et al., 2013). Bereavement or severe illness in a close relative during the pre-conception period can also increase the risk of preterm birth (Khashan et al., 2009), which is independently associated with atypical neurodevelopment (Johnson and Marlow, 2017).

It should be noted there are 2 components which dictate the response to trauma; objective hardship and subjective stress. Both of which disproportionately affect those in lower socio-economic brackets (Mcewen and Gianaros, 2010; Ursache et al., 2015). These risk factors overlap with those for chronic maternal distress, but subjective stress is derived from genetic components which are distinct from instances of chronic distress, such as depression (Rietschel et al., 2014).

### Maternal Distress

Anxiety and depressive disorders are pervasive throughout society but women, particularly during their reproductive years, are more likely to be affected (Seedat et al., 2009; Parsons et al., 2012). Of particular note is that anti-depressant use during pregnancy has been associated with adverse birth outcomes (Huang et al., 2014) and altered brain connectivity in offspring (Lugo-Candelas et al., 2018). However, it should be noted this is not seen in all studies (El Marroun et al., 2014). Nonetheless, many women go untreated for depression during pregnancy, likely contributing to a more severe psychopathology (Geier et al., 2015; Byatt et al., 2016). Data from the Avon Longitudinal Study of Parents and Children (ALSPAC) show that maternal anxiety and depression are associated with persistent and clinically meaningful alterations in behavior and emotionality of offspring (O'Donnell et al., 2014), as well as psychotic experiences by 18 years of age (Srinivasan et al., 2020). ALSPAC analyses reveal that the well-established association between maternal depression with that for the offspring (Weissman et al., 2006) is largely attributable to the pre-natal maternal condition (Pearson et al., 2013). The association between maternal depression and cognitive outcomes in the offspring shows the same pre-natal dominant influence (Evans et al., 2012).

Meta-analyses indicate an incidence of 15.2% for any anxiety disorder (Dennis et al., 2017) and 7.4-12.8% for depression (Bennett et al., 2004) during pregnancy. It is also likely these numbers are an underestimation, as a significant portion of mood disorders during pregnancy go undiagnosed (Dietz et al., 2007; Ko et al., 2012). Perinatal mental health disorders are also more common in lower and middle income countries (Fisher et al., 2012), likely a result of the social and economic factors which may influence chronic stress during pregnancy. In contrast, the incidence of common drivers of maternal trauma, death or diagnosis with a serious medical condition in a close relative, ranges from 1.6 to 2.7% (Khashan et al., 2009; Class et al., 2013). Therefore, a better understanding of the fetal effects of chronic distress is, unequivocally, an important public health issue.

### Animal Models

Considering the inevitable confounders present in human cohorts (i.e., co-morbidities and socio-economic factors), ascribing a specific event or perturbation to a subsequent outcome can be difficult. To this end, animal models have been fundamental to the establishment of a causal relationship between pre-natal stress and behavioral outcomes. Initial animal models used conditioned aversive stimuli to induce distress in pregnant rodents (Thompson, 1957), with subsequent experiments using a variety of physical and sensory stimuli including physical restraint (Ward, 1972) and electric shocks (Takahashi et al., 1998). Many stimuli can be used to elicit distress in rodents including; restraint, bright light, housing in a small cage, white noise, predator noises or scents, sleep deprivation, forced swimming or exposure to a lactating female (reviewed thoroughly in Weinstock, 2017). Chronic variable stress (CVS) is a common model of pre-natal stress and uses combinations of these stimuli, staged at random points during the day to avoid habituation. These models are typically only used for a period of pregnancy (~1 week) and therefore are not designed to investigate chronic distress, associated with persisting adverse mental health, but rather the timing and critical periods associated with acute pre-natal stress exposure. This feature of the experimental design is unquestionably important to understand, but as we will discuss later, during pregnancy, maternal distress is more likely to be driven by chronic stressors that remain stable over longer periods of time, to which current CVS models have less obvious parallels. Considering social stress is likely to have a central role in instances of human chronic distress, the clear bias toward physical stressors is also a potential limitation in these studies. Future studies may also consider the inclusion of social stressors in these paradigms (e.g., social defeat or isolation).

Models akin to CVS are used to induce depressive and anxiety-like behaviors in non-pregnant adult rodents (Lezak et al., 2017), and do recapitulate gene expression patterns associated with human depression (Scarpa et al., 2020). However, these depression models are used for several weeks (Wang et al., 2017) and variability in results using this model has been ascribed to shorter paradigm durations (Willner, 2017), indicating a longer period of exposure is needed to reproducibly induce depressive and anxiety-like behaviors in adult animals. Importantly chronic stress exposure in pregnant mice results in anxiety and depressive like behaviors in dams (Salari et al., 2016).

### Behavioral Effects in Animal Models

Early studies showed an increased anxiety-like phenotype in pre-natally stressed rats using the elevated plus maze (Fride and Weinstock, 1988). This finding has subsequently been confirmed in several labs using both sexes and multiple strains of both rats and mice (Walf and Frye, 2007; Akatsu et al., 2015; Palacios-García et al., 2015; Zohar et al., 2015). An increase in depressive-like behaviors measured using the forced swim test and sucrose preference test, has also been seen in both sexes and various strains of rat following pre-natal stress (Abe et al., 2007; Butkevich et al., 2011; Guan et al., 2013; Sickmann et al., 2015; Weinstock, 2017). Alterations in social behaviors, spatial memory, novel object recognition, addictive behaviors and sexual activity have also been described following pre-natal stress in rodents (Holson et al., 1995; Markham et al., 2010; Lui et al., 2011; Salomon et al., 2011; Wilson and Terry, 2013; Barzegar et al., 2015; Dong et al., 2018; Gur et al., 2019). Also similar to data in humans, the temporal onset of stress during pregnancy is associated with different cognitive, metabolic and weight outcomes (Mueller and Bale, 2006, 2007). However, despite dramatic advances in technology and methodology in recent times, there has been relatively little novel insight into the mechanistic underpinnings of these behavioral alterations.

## Summary and Perspective

A number of excellent reviews (e.g., Monk et al., 2013; Glover, 2014; O'Donnell et al., 2014; Bronson and Bale, 2016) underscore the importance of studies that use model systems in establishing the impact of pre-natal maternal health on offspring development. However, the role for “basic science” studies of pre-natal maternal stress and mental health has remained surprisingly descriptive, with little attention dedicated to the study of biological mechanisms. Despite novel insight from several studies in non-human species (e.g., Gapp et al., 2014; Jašarević et al., 2018; Nugent et al., 2018; Bittle et al., 2019; Jašarević and Bale, 2019), our understanding of candidate mechanisms involved in pre-natal stress remains poor. Furthermore, studies of pre-natal maternal stress in non-human species have largely failed to integrate major advances in the biological sciences, such as single-cell sequencing methods, CRISPR-Cas9 based genetic manipulation and brain organoids. These techniques and systems are powerful methods for the identification and evaluation of novel mechanisms, which need to be embraced by the field moving forward. Indeed, perhaps the most important research concerning mechanisms for the influence of maternal mental health conditions on offspring neurodevelopment is that which imply mediation by inflammatory signals, which derives almost exclusively from human studies (Hantsoo et al., 2019). Likewise, neuroimaging studies with children have contributed more to our understanding of neural circuits, the development of which is vulnerable to the influence of pre-natal maternal mental health, than has the rather dated neuroanatomical evidence from model systems (see Charil et al., 2010 for a review). We suggest that technological and computational advances in the biological sciences can contribute enormously to our understanding of maternal influences on fetal neurodevelopment. However, an equally important consideration is the need to better align our experimental models of pre-natal maternal stress to the relevant human condition and associated health outcomes. Here, we consider both of these issues for the future of neuroscience research on the topic of pre-natal stress, beginning with the more fundamental issue of alignment.

## Aligning Studies of Pre-natal Stress in Model Systems With the Human Condition

We suggest that by their very design, the vast majority of research using non-human models best aligns with the maternal trauma stream of human epidemiological research. In these studies, stress is imposed on pregnant females usually at specific time periods over pregnancy. It is common that such studies are expressly designed to address issues related to “critical periods”; when in the course of pregnancy are specific developmental outcomes most likely to be affected by maternal stress? The same issue is often addressed in the analysis of human studies of maternal stress. Our argument is not that the issue of critical periods is irrelevant for studies of maternal influences on neurodevelopment, but rather that the relevance is greater for some models, less so for others. Furthermore, we acknowledge that mechanisms and critical periods identified using existing animal models may also have relevance to instances of more chronic exposures.

The issue of critical periods has a long history in neuroscience and is important for our understanding of the effects of maternal trauma. Such studies may elucidate not only the magnitude of the effect but also the nature of the neurodevelopmental impact. Timing matters. However, this focus has, in our opinion, led to experimental research models that ignore the prevalent human reality of chronic forms of maternal adversity subsumed under our term of maternal distress. The relevant human conditions of “distress” are those of women with high sub-clinical or clinical levels of depression or anxiety and/or chronically elevated perceived stress. In each of these instances, the relevant maternal condition operates throughout the pregnancy. In contrast to the impression engendered by the regrettable term “post-partum depression,” the measures that capture the various forms of maternal distress reveal a striking level of stability over the perinatal period. Longitudinal trajectory analyses reveal that depressive symptom levels are largely stable over the pre- and post-natal periods (Santos et al., 2017; Lim et al., 2019) with similar profiles for perceied stress (Lim et al., 2020). Analyses of the ALSPAC cohort reveals modestly higher depressive symptom levels in early pregnancy (Evans et al., 2001; Heron et al., 2004), with a similar trend for symptoms of anxiety (Heron et al., 2004). Latent class analyses and other forms of statistical modeling with longitudinal cohort data show that most women exhibit stable low, moderate or high symptom levels of depression (Cents et al., 2013; Giallo et al., 2014; Van Der Waerden et al., 2015; Park et al., 2018 and see Santos et al., 2017 for review). Only a smaller percentage of women show marked increases in symptom levels that reach clinical levels in the post-partum period, thus revealing a post-partum onset of clinical symptoms. However, there are mixed reports on the stability of anxiety levels during pregnancy and post-partum. Whilst most studies observe lower levels of anxiety after delivery than during pregnancy (Heron et al., 2004; Figueiredo and Conde, 2011), others reveal no difference in anxiety levels (Grant et al., 2008). Therefore, while some women do experience a post-partum onset of depression, the evidence suggests that most women generally report stable levels of depressive and anxiety symptoms over the peripartum period. The trend for perceived stress reveals even stronger evidence for peripartum stability (Lim et al., 2020). In sum, the evidence from longitudinal studies of human mothers reveals a pattern of stability, which implies that the relevant maternal mental health conditions are chronic.

The relative stability of maternal symptoms of depression and anxiety is not surprising since the strongest predictor of post-natal depression is depression during pregnancy (O'Hara and Swain, 1996; Llewellyn et al., 1997; Leigh and Milgrom, 2008). The origins of clinical or high, sub-clinical maternal symptoms of depression and anxiety may extend even further, into the pre-conception period. Marcus et al., 2003 found that almost half of the women with depression during pregnancy had a history of major depressive disorder. Indeed, the most important risk factor for perinatal depression is a prior history of depression (Leigh and Milgrom, 2008; Giallo et al., 2014; Patton et al., 2015). Reviews of psychosocial risk factors for perinatal depression reveal strong evidence for a lack of social support, domestic violence, perceived stress, adverse life events and low socio-economic status (Lancaster et al., 2010; Yim et al., 2015; Biaggi et al., 2016; Santos et al., 2017). These factors are typically stable and also predict levels of depression and anxiety in the general population (Kessler et al., 2003). Stable personality traits such as neuroticism that predict anxiety and depression in the general population, also predict the risk for poor maternal mental health (Leigh and Milgrom, 2008; Martini et al., 2015; Denis and Luminet, 2018). Protective factors such as social support and self-esteem that diminish the risk for depression and anxiety in the general population show a comparable influence on peripartum depression and anxiety (Elsenbruch et al., 2007; Leigh and Milgrom, 2008; Martini et al., 2015).

Since conditions that predict poor maternal mental health in pregnancy pre-date conception, it is unsurprising that so do levels of depression and anxiety. For many women the relevant maternal condition was present prior to pregnancy and thus predated conception. Perinatal mental health problems are commonly preceded by problems prior to conception, often during adolescence (Patton et al., 2015). Kee et al. (2021) showed that for two-thirds of mothers in a longitudinal cohort with clinical or high, sub-clinical levels of depressive symptoms, the mental health condition was apparent in the pre-conception period. A prospective and transgenerational Australian study provides daunting evidence for the importance of the pre-conceptual period, showing that pre-conception levels of depression predicted emotional reactivity of the offspring independent of maternal depression in either the antenatal or post-natal period (Spry et al., 2020).

## Implications for Studies With Model Systems

Studies using model systems inspired by associations observed in humans between the various measures of chronic maternal distress and the risk for affective disorders in the offspring (i.e., “internalizing” forms of psychopathology) must consider the nature of the relevant maternal influence in humans. Longitudinal trajectory analyses clearly show that maternal distress, which predicts an increased risk for depression and anxiety as well as multiple cognitive outcomes in the offspring, is highly stable over the perinatal period. Moreover, antenatal symptoms of depression and anxiety are often consistent with those in the pre-conception period. Thus, the clinically relevant model for the effects of maternal distress on the subsequent risk for depression or anxiety is one that imposes stress on dams over the entire period of pregnancy, including the pre-conception period. Ideally such models would also extend into the post-partum period where the relevant research objectives do not hinge on the timing of effects. Post-partum measures of relevance could involve maternal care, which is affected by pre-natal stress in rodents (Champagne and Meaney, 2006). We acknowledge that this design complicates the issue of timing. However, it is our opinion that these issues can be effectively addressed with more ambitious research designs and discuss this issue later in this paper. The plea is straightforward: research addressing the mechanisms for the association between maternal distress and offspring neurodevelopment and health must respect the chronicity of the relevant maternal conditions in the human condition.

## The Prevalence of Maternal Distress

There is a long and impressive scientific literature on the effects of clinical states of maternal depression or anxiety on neurodevelopmental outcomes in the offspring (e.g., Weissman et al., 2006; Monk et al., 2013; Glover, 2014; O'Donnell et al., 2014). Such studies commonly employ case: control analyses of mothers with confirmed clinical diagnoses. These studies document the powerful associations between clinical states of maternal depression and anxiety, and a wide range of neurodevelopmental and health outcomes in the offspring. An obvious issue is whether effects are unique to those women with clinical levels of symptoms. Studies addressing this issue reveal the considerable impact of high, sub-clinical symptom levels of depression (see Meaney, 2018 for review). The psychosocial function and parenting of women with high, sub-clinical levels of depressive symptoms is as affected as are those with clinical levels of depressive symptoms (Judd et al., 1996; Lovejoy et al., 2000; Weinberg et al., 2001). Estimates of the impact of maternal mental health problems based on clinical cut-offs or diagnosis thus underestimate the extent of the problem across the population. In higher income countries, the prevalence of clinical levels of maternal depression is commonly between 10 and 15% (Bennett et al., 2004; Gavin et al., 2005; Wachs et al., 2009; Ertel et al., 2011). The distribution of depressive symptoms across populations is normal and unimodal such that there are 2–3 times more women with high, sub-clinical levels of symptoms compared with those with clinical levels. In economically developed countries, mothers with clinical and high-subclinical levels of depressive symptoms include as much as 40% of the population, thus representing a public health issue of remarkable importance (Meaney, 2018). Moreover, while less well-studied because of the lack of established clinical cut-offs, the prevalence rates of perinatal maternal anxiety appear even higher than those for depression, and consequences for the development of the offspring are at least as impactful as are those of depressive symptoms (van Batenburg-Eddes and Jolles, 2013; Glover, 2014; O'Donnell and Meaney, 2017; Dean et al., 2018; Madigan et al., 2018).

More recent studies underscore the equally pronounced associations between levels of maternal stress and child outcomes (Monk et al., 2013). The influence of these studies has resulted in longitudinal, prospective birth cohort studies with community samples that examine symptoms of depression, anxiety and perceived stress across a continuum. Studies of developmental outcomes in the offspring support a uniform conclusion: The measures of maternal distress operate across a continuum to influence neurodevelopmental outcomes (Meaney, 2018). Neuroimaging as well as measures of cognitive - emotional function in the offspring reveal a graded influence of maternal distress across the normal range of values for measures of maternal distress. Studies of the “unaffected” offspring (i.e., those without depression at the time of testing) of mothers with clinical levels of depression, compared to those of mothers without diagnosed mental disorders, reveal differences in hippocampal and/or amygdala volume, the neural circuitry associated with reward processing and cortical thickness (Chen et al., 2010; Gotlib et al., 2010; Lupien et al., 2011; Peterson and Weissman, 2011). Variation in each of these measures has been observed in community samples that examine symptoms of depression or anxiety across the normal range (Rifkin-Graboi et al., 2013; Qiu et al., 2015; Sandman et al., 2015; Lebel et al., 2016; Wen et al., 2017 and reviewed in O'Donnell and Meaney, 2017; Meaney, 2018). An impressive analysis of cortical thickness (Sandman et al., 2015) yielded a significant association with maternal depressive symptoms despite the almost complete absence of cases that would pass the clinical screening cut-off for maternal depression. Likewise measures of both socio-emotional and cognitive outcomes in childhood show a graded association with maternal symptoms of depression or anxiety, as well as across levels of perceived stress (Evans et al., 2012; Cents et al., 2013; Yan and Dix, 2016 and see Supplementary Material in Meaney, 2018 for a systematic review). An extensive review (Goodman et al., 2011) reveals that the association between symptoms of maternal depression and internalizing and externalizing problems during childhood is apparent in both community studies as well as in case: control analyses. This spectrum-like association we describe provides strong evidence for causation, which is analogous to the importance of a dose-response relationship in the assignation of causation in toxicology studies.

## The Use of Model Systems to Study Phenotypic Dimensions

The compelling evidence for the influence of maternal distress across a continuum, as opposed to uniquely clinical conditions, has important implications for studies with model systems. A commonly cited limitation of studies of mental health using model systems is the inability to define disease states in non-human species, and thus establish “face validity.” The RDoC (Research Domain Criteria) initiative (Insel et al., 2010; Casey et al., 2013) has mitigated this concern shifting the emphasis from clinically diagnosed syndromes to specific phenotypic dimensions associated with variations in mental health status. This dimensional perspective permits measures to be considered along a continuum applicable to the study of mental health, broadly defined, as opposed to disease. This approach moves the challenge of face validity to that of specific phenotypes, as opposed to complex, multi-dimensional clinical syndromes. Altered patterns of sleep, reward sensitivity, attention, stress reactivity, etc. are features of depressed and/or anxious conditions and disrupted by chronic stress. Each can be assessed in model systems.

Analyses of associations between various measures of maternal distress and child outcomes reveal effects across a continuum, which in our opinion strengthens the case for the use of model systems in which specific maternal phenotypic dimensions can be associated with outcomes in the offspring. One approach that would further strengthen the relevance of studies with non-human species is to relate the stressor to specific features of the maternal condition and outcomes in the offspring. A strength of this approach is that it would allow comparative analyses at the level of specific features of the maternal condition and associations with developmental outcomes in the offspring that would benefit our depth of understanding and inform intervention programs. This approach bears considerable potential to inform intervention studies in humans by identifying the feature of the maternal condition most relevant for the specific offspring outcome. As an example, more fearful female rats exhibit reduced post-partum levels of pup licking/grooming (Champagne and Meaney, 2001) with “downstream” effects on cognitive – emotional outcomes in the offspring (Meaney, 2001). Stress imposed on pregnant, high-licking mothers increases fearfulness eliminating the differences in fear behavior as well as in maternal behavior between high and low pup licking/grooming rats (Champagne and Meaney, 2006). The effect of pre-natal stress on fearfulness mediates the effect on maternal behavior and the subsequent influence on developmental outcomes in the offspring. Environmental enrichment produces exactly the opposite transformation by decreasing fearfulness in low pup-licking females (Champagne and Meaney, 2007). The effect is best understood as an influence of maternal fearfulness and not pre-natal stress, *per se*. Pre-natal stress is the distal influence; maternal fearfulness the proximal, dimensional factor. As noted above, there are multiple functional dimensions that link perceived stress to maternal health in humans that can be approximated in other animals.

## Maternal Distress: A Consideration of Mediators, Moderators and Mechanisms

Maternal distress during pregnancy confers an increased risk for a psychiatric disorder diagnosis in offspring. The resulting disorder are typically diagnosed in later life with relatively few novel therapeutic options available (Nestler and Hyman, 2010). A better understanding of the pre-natal risk factors and the mechanisms through which they act is likely central to the development of novel therapeutic strategies. To date, mechanistic investigation into the consequences of maternal distress have largely focused on the traditional central mediator of the acute stress response, the hypothalamic-pituitary-adrenal (HPA) axis. This focus has provided valuable insights into how maternal distress can shape psychiatric risk, but it is now clear that alterations within the HPA axis cannot account for the entire spectrum of risk (O'Donnell and Meaney, 2017). We suggest a more diverse investigation of the mechanisms involved in maternal distress, using advanced techniques, is necessary to understand this increased risk. In particular novel -omics approaches [for a full review of -omics based methods and their limitations see Hasin et al. (2017) and Karczewski and Snyder (2018)] offer insight at an unprecedented resolution. Herein, we discuss candidate mediators, moderators and mechanisms, along with the existing evidence for their interaction with maternal distress and psychiatric risk. We make reference to human data where available and draw insight from existing animal models of maternal trauma where appropriate. However, in line with the central thesis of this review, we acknowledge future animal studies of chronic maternal distress are necessary for a comprehensive understanding of the mediators, moderators and mechanisms associated with psychiatric risk.

### The HPA (Hypothalamic-Pituitary-Adrenal) Axis

In humans, HPA axis function and stress reactivity in children can be affected by maternal anxiety, depression (O'Donnell et al., 2013; Capron et al., 2015) or maternal trauma (Yong Ping et al., 2015), with alterations in diurnal cortisol rhythms found to persist until adolescence following pre-natal exposure to maternal anxiety or depression (O'Donnell et al., 2013). In non-human primates (Coe et al., 2003; Murray et al., 2018), guinea pigs (Kapoor and Matthews, 2005, 2008) and rodents (Brunton and Russell, 2010; St-Cyr et al., 2017) exposure to maternal distress has been associated with sex-specific alterations within the HPA axis of the offspring. This effect is also present in several other divergent species, suggesting pre-natal programming of the HPA axis is a highly conserved mechanism (Thayer et al., 2018).

Exogenous administration of synthetic or endogenous glucocorticoids (i.e., dexamethasone and cortisol/corticosterone, respectively), are commonly used to model glucocorticoid excess during pregnancy in a variety of animal models, with exposure associated with a variety of deficits. For instance, pre-natal exposure to dexamethasone in rats produces anxiety-like behaviors in females only (Hiroi et al., 2016), and a reduction in proliferation of neural progenitor cells (Samarasinghe et al., 2011). Using human forebrain organoids expression of genes implicated in psychiatric disorders have also been shown to be affected by dexamethasone exposure (Cruceanu et al., 2020).

However, neither plasma corticotropin-releasing factor (CRF) or hair cortisol are correlated with maternal pregnancy-related anxiety (Kramer et al., 2009; Orta et al., 2018). Salivary cortisol has been associated with anxiety during pregnancy, but only after the 30th week of gestation (Kane et al., 2014) and structural alterations within the fetal brain associated with maternal distress can already be seen by this timepoint (Wu et al., 2020). While in both pregnant and non-pregnant individuals, cortisol is not reliably associated with depression (Seth et al., 2016; Nandam et al., 2020). Therefore, while glucocorticoids demonstrably influence brain development, their contribution to the increased psychiatric risk associated with maternal distress is unclear. Likewise, the follow-up studies of children exposed to therapeutic doses of synthetic glucocorticoids during fetal life has produced equivocal findings using a variety of neurodevelopmental outcome measures (reviewed in O'Donnell and Meaney, 2017).

### Allopregnanolone

Allopregnanolone is another secreted steroid (a derivative of progesterone) with well-described signaling functions in the brain (Paul et al., 2020). Maternal plasma concentrations of allopregnanolone increase dramatically during pregnancy (Kancheva et al., 2007) and allopregnanolone is responsive to both acute and chronic stress (Bali and Jaggi, 2014). Pre-natal maternal distress in rats is associated with sex-specific changes in the distribution of allopregnanolone within the offspring brain (Torgersen et al., 2020). Considering allopregnanolone is a strong modulator of GABA (gamma-Aminobutyric acid) signaling (Carver and Reddy, 2013), this may have important implications for circuits throughout the brain.

Allopregnanolone administration can normalize HPA axis reactivity following pre-natal exposure to maternal social stress in female rats (Brunton et al., 2015). Allopregnanolone is also decreased in the brain of individuals with depression (Uzunova et al., 1998; Schüle et al., 2006), as well as in non-human animal models of depression (Almeida et al., 2020). However, a study of 284 pregnant women did not find an association between plasma allopregnanolone and depression at the 18^th^ week of gestation (Hellgren et al., 2017). Although, another study found that allopregnanolone levels measured in the second trimester were associated with an increased risk of post-natal depression (Osborne et al., 2017). In line with the RDoC initiative outlined earlier, allopregnanolone should be characterized with respect to specific symptoms and imaging phenotypes to clarify its role in maternal distress. Nevertheless, the protective effects of allopregnanolone in non-human models following pre-natal stress (Brunton et al., 2015) warrant further investigation.

### Resiliency

A large proportion of individuals exposed to pre-natal maternal distress do not develop a psychiatric disorder. This concept of resiliency is more commonly studied in non-pregnant adult animals (Russo et al., 2012) but it is unclear how well these findings relate to pregnancy considering the distinct social and physiological environments involved. Moreover, there are a complex set of interactions between maternal genetics and environment, and subsequently fetal genetics and environment which need to be considered. To understand psychiatric risk in offspring, the nature of resilience at each of these levels must be characterized. For instance, maternal resilience to the psychiatric correlates of chronic distress, may be mediated through genetic mechanisms. This does not preclude elevations in blood borne signaling molecules associated with distress which may entrain the fetal brain, thereby maintaining fetal exposure and elevating fetal risk in the absence of maternal symptoms of distress. Moreover, there are an increasing number of loci associated with resilience (Maul et al., 2020), underlining the importance of understanding the polygenic component of resilience and its associated mechanisms. It should also be noted that resiliency is multi-factorial in nature and also influenced by maternal behaviors (Bolten et al., 2012; Brotnow et al., 2015). Further, pre-natal stress can affect subsequent responses to the post-natal environment through plasticity related mechanisms (Hartman and Belsky, 2018).

### Polygenic Risk

A major difficulty in human studies which assess psychopathology risk following instances of maternal trauma or distress is distinguishing the relative contribution of genetic and environmental components. The increasing size of human cohorts and genetic analyses have given clear demonstrations of the polygenic risk associated with psychiatric disorders. These studies also indicate large degrees of genetic pleiotropy and indicate relevant genes are highly expressed during fetal development (Cross-Disorder Group of the Psychiatric Genomics Consortium et al., 2013, 2019). Furthermore, this polygenic component can interact with the environment to modulate outcome. For instance, Qiu et al. (2017) reported that a polygenic risk score for major depressive disorder moderated the association between pre-natal maternal symptoms of depression and hippocampal and amygdala volume assessed with neonatal magnetic resonance imaging (MRI). The interaction between this polygenic component and the environment is key to understanding absolute risk, but this polygenic risk cannot currently be accurately modeled in non-human models. In recent years, brain organoid models have become a reproducible and accessible human model of early neurodevelopmental processes (Camp et al., 2015; Yoon et al., 2019). They provide an excellent opportunity to model the interaction between polygenic risk and environmental perturbations or signaling mediators, on relevant neurodevelopmental processes.

### Epigenetics

Epigenetic marks, such as DNA methylation and histone modifications, are also important factors to consider. They are dynamically present throughout neurodevelopment (Spiers et al., 2015; Amiri et al., 2018) and likely have a role in determining psychiatric outcomes (Kuehner et al., 2019). Sexual diversification during brain development also heavily relies on epigenetic mechanisms (McCarthy et al., 2017) and as such may contribute to sex-related differences in psychopathology following maternal distress. In humans, changes in DNA methylation within cord blood and blood during childhood have been associated with pre-natal maternal distress (Cao-Lei et al., 2014; Wu et al., 2018). Similarly, animal models of maternal distress have demonstrated changes in DNA methylation and histone modifications in the placenta and fetal brain (Peña et al., 2012; Matrisciano et al., 2013; Benoit et al., 2015; Nugent et al., 2018).

Much of this work has been carried out using candidate methods but epigenetic alterations at a single locus are unlikely to offer robust and novel mechanistic insight. Genome wide approaches are now generally affordable with well-described analysis pipelines (Yong et al., 2016; Cuvier and Fierz, 2017) and should be further embraced to generate novel insight. Furthermore, most of this work has been correlational in nature due the lack of tools to specifically modify the epigenome. Recently with the revolution in CRISPR-Cas9 genome editing technology, a range of epigenetic modifying tools have become available (see Yim et al., 2020 for review). These tools allow for the specific manipulation of an epigenetic mark, to identify causal mechanisms for relevant psychiatric phenotypes. These methods will be central for future studies to examine the ability of epigenetics to mediate outcomes following maternal distress.

### Circadian Rhythmicity

Circadian rhythms are present in biology from the behavioral (i.e., sleep-wake cycles) down to even the epigenetic level (Etchegaray et al., 2003; Azzi et al., 2014), and changes in circadian rhythmicity are a central component of depression and anxiety disorders (Germain and Kupfer, 2008; Walker et al., 2020). Circadian disruption during pregnancy in the form of a fixed night shift schedule is associated with an increase in fetal loss during the late stages of gestation (Zhu et al., 2004) and other adverse pregnancy outcomes (Cai et al., 2019). In animal models, constant light is associated with altered gestational length (Mendez et al., 2016; Gatford et al., 2019), along with altered circadian rhythms (Ohta et al., 2006) and spatial memory (Vilches et al., 2014) in offspring. It is not clear whether this effect is moderated by maternal psychological distress, but gestational restraint stress in mice alters circadian gene expression in the suprachiasmatic nucleus of offspring (Yun et al., 2020) and pre-natal maternal anxiety in humans is associated with sleep problems in offspring at 18 and 30 months (O'Connor et al., 2007).

Mutation of the core circadian regulators *Clock* or *Per1* and *Per2* in mice results in anxiety-like behaviors (Roybal et al., 2007; Spencer et al., 2013). *Clock* is involved in the regulation of critical periods during neural circuit maturation (Kobayashi et al., 2015) and *Per1* is a well-characterized stress response gene (Al-Safadi et al., 2015). However, the function of circadian patterns of gene expression during typical brain development requires further study in order to establish their role in anxiety- and depressive-like behaviors. The development of novel single cell analysis methods and genetic models (Fonseca Costa et al., 2020; Shan et al., 2020; Wen et al., 2020) offer the opportunity to study these effects at unprecedented resolution and will likely play a central role in these future investigations.

Furthermore, several key maternal hormones are secreted in circadian patterns. For instance melatonin, which can readily cross the placenta (Naitoh et al., 1998) and has neuro-protective effects in humans and animal models following inflammation and asphyxiation (Fulia et al., 2001; Welin et al., 2007; Castillo-Melendez et al., 2017; Aridas et al., 2018). The melatonin receptor is expressed in many nuclei throughout the fetal brain including many within the hypothalamus (Thomas et al., 2002) and circulating nocturnal melatonin is decreased during pregnancy but increased in the post-partum period in women with depression (Parry et al., 2008). Interestingly, maternal melatonin can modulate fetal cortisol levels in non-human primates (Torres-Farfan et al., 2004), but the dynamics of melatonin signaling in the fetal brain during maternal distress and their consequences for subsequent outcomes are largely unknown. Many key hormones of the HPA (hypothalamic-pituitary-adrenal) axis are also secreted in a circadian fashion, with changes seen in their secretion in offspring exposed to maternal distress (O'Donnell et al., 2013).

### Extracellular Vesicles (ECVs)

Another form of maternal-fetal communication occurs through ECVs, which are small membrane enclosed bodies which all cells are capable of producing. They can contain nucleic acids, proteins or metabolites and play an important role in cell-cell signaling in a number of contexts, including maternal-fetal signaling (Van Niel et al., 2018; Buca et al., 2020). ECVs are involved in signaling pathways associated with depression (Brites et al., 2015) and can affect neurodevelopment in offspring following paternal distress through pre-conception signaling mechanisms in sperm (Chan et al., 2020). However, the role of ECVs in maternal-fetal signaling during maternal distress remains largely unknown. Deep RNA sequencing and global proteomic approaches enable an unbiased analysis of ECV cargo in both humans and animal models (Huang et al., 2013; Choi et al., 2015) and these techniques will be important in establishing the role of ECVs in maternal-fetal signaling during distress. This is particularly true for processes, such as inflammation, which have frequently been shown to use ECV signaling mechanisms (Słomka et al., 2018; Van Niel et al., 2018).

### Inflammation

Both human and non-human studies have described a central role of inflammatory mechanisms in depression (Slavich and Irwin, 2014; Miller and Raison, 2016). A wealth of data also indicate inflammatory mechanisms may, at least in part, mediate the effects of maternal distress on the fetus (see Hantsoo et al., 2019 for review). Considering perinatal inflammation is a potent modifier of neurodevelopment and offspring behavior (Hagberg et al., 2015; Choi et al., 2016; Reed et al., 2019), the role of inflammatory mechanisms in this capacity have been understudied.

Maternal distress in humans has been associated with altered levels of plasma inflammatory mediators throughout pregnancy (Coussons-Read et al., 2007; Blackmore et al., 2011; Walsh et al., 2016; Gilman et al., 2017) and in rodents pre-natal stress is associated with elevated cytokines and altered microglial phenotypes in the neonatal brain (Diz-Chaves et al., 2012; Slusarczyk et al., 2015; Chen et al., 2020). In adult mice, peripheral immune cells can regulate anxiety-like behaviors through IL-17 (Alves de Lima et al., 2020) and maternal IL-17 has been shown separately to modulate neurodevelopment and social behavior in mice exposed to an inflammatory stimulus (Choi et al., 2016; Reed et al., 2019). Furthermore, in mice IL-6 has been shown to drive phenotypic alterations in microglia within the embryonic cortical plate following pre-natal stress (Gumusoglu et al., 2017). Intriguingly, research suggests that pre-natal administration of non-steroidal anti-inflammatory drugs can ameliorate some of the symptoms associated with a pre-natal CVS model (Bronson and Bale, 2014). Understanding the mediators and signaling pathways involved in these effects are important considerations for future studies. Recently, large reference datasets for the automatic annotation of peripheral immune cells following single cell sequencing (Hao et al., 2020) have been developed, which could be used in future studies alongside global proteomic approaches in both the maternal and fetal compartments to identify maternal signaling mediators involved in the conferral of risk for psychopathology.

### Placental Dysfunction

Considering the central role of the placenta in maternal-fetal exchange, it is a prime candidate to mediate the *in-utero* effects of maternal distress including those from inflammatory mediators. However, it is surprisingly understudied in this context, despite insightful results in animal models. For instance, in a pre-natal CVS model, placental expression of *O-GlcNAc transferase* (*OGT*) has been described as a key moderator of fetal growth and HPA axis functionality (Howerton and Bale, 2014) and placental H3K27me3 has been shown to be involved in establishing sex specific responses to CVS (Nugent et al., 2018). The enzyme 11β-HSD2 which converts glucocorticoids to their active or inactive form at the placenta, thereby controlling fetal glucocorticoid exposure, is also affect by maternal anxiety in humans (O'Donnell et al., 2012).

The placenta is unique in that it can be sampled and examined using -omics methodologies with little physiological intrusion to the mother or child. Preliminary studies have taken advantage of this by using arrays to identify gene expression changes within the placenta associated with maternal depression (Olivier et al., 2015; Edvinsson et al., 2019). The more recent approach of single cell sequencing has been used in human placenta tissue to identify changes associated with preeclampsia and preterm birth (Tsang et al., 2017; Pique-Regi et al., 2019), and a similar approach to delineate cell specific gene expression and epigenetic states in the placenta of pregnancies associated with anxiety or depression would be extremely informative.

### Microbiome

Similarly to the placenta the microbiota can be examined in both the mother and fetus with relatively little intrusion and in recent years the sensitivity of the microbiome to the environment and its importance in brain development has become appreciated. For instance, in adult animals the microbiome mediates the behavioral effects of stress (Marin et al., 2017; Pearson-Leary et al., 2020) and in humans, probiotics during pregnancy have proven effective in alleviating post-natal symptoms of depression and anxiety in mothers (Barthow et al., 2016; Slykerman et al., 2017). This is particularly pertinent as the maternal microbiome is a crucial mediator of typical fetal brain development (Vuong et al., 2020). In rodents, pre-natal stress can drive changes in offspring microbiota composition and the intestinal transcriptome (Jašarević et al., 2018), while in humans microbiota changes within the meconium have been associated with maternal anxiety (Hu et al., 2019). The dynamics of these changes and their involvement in mediating psychiatric risk will be important future considerations. In recent proof-of-concept work, fetal microbiota composition was demonstrated to be amenable to manipulation through fecal microbiota transplantation (Korpela et al., 2020), which may point to a novel therapeutic strategy if microbiota are demonstrated to mediate psychiatric risk associated with pre-natal exposure to maternal distress.

### Neurogenesis and Neuronal Maturation

A wealth of human MRI data points to both structural and functional changes within the brain of children following maternal distress (see Lautarescu et al., 2020a for review). Structural and micro-structural changes have also been described in the neonatal period (Li et al., 2012; Rifkin-Graboi et al., 2013; Qiu et al., 2015; Lautarescu et al., 2020b) and *in utero* (Wu et al., 2020). Processes such as neurogenesis and gliogenesis are obvious candidates to mediate these early changes, and indeed a variety of models, across different species and brain regions, have demonstrated altered neurogenesis (Coe et al., 2003; Kraszpulski et al., 2006; Rayen et al., 2011) and dendritic spine distribution (Murmu et al., 2006; Bock et al., 2011; Petit et al., 2015) in the neonatal and adolescent period following pre-natal maternal distress. Interneuron maturation and migration is also affected by pre-natal maternal distress in rodents (Fine et al., 2014; Lussier and Stevens, 2016), which may be of particular interest considering the expanded program of interneuron generation and migration seen specifically in humans (Paredes et al., 2016). As such, changes in neurogenesis and neuronal maturation are candidates to underlie the structural alterations seen in humans following maternal distress, but further work is needed to understand the signaling pathways and cellular dynamics associated with these effects. Furthermore, very few studies have leveraged MRI techniques to investigate changes associated with maternal distress in model systems, despite the widespread availability of the hardware required for image acquisition. Considering the routine use of MRI in human studies, MRI studies with mammalian model systems would provide an elegant comparison without the inevitable confounders associated with human studies.

## Conclusion

In this review we highlight important considerations for the future of pre-natal stress research. We indicate that the majority of pre-clinical work aligns with instances of maternal trauma and, while acknowledging the importance of this work, we suggest the prevalence of anxiety and depressive disorders amongst pregnant women dictates a higher priority within non-human models. We suggest existing models of maternal distress, such as the chronic variable stress model, should be expanded to all of gestation and the pre-conception period, a timeline which is analogous to the relevant human risk factors. Considering the limited novel mechanistic insights into the underpinnings of psychiatric risk, we also suggest a more diverse range of mechanisms need to be investigated using advanced techniques and analysis methods.

## Data Availability Statement

The original contributions presented in the study are included in the article/supplementary material, further inquiries can be directed to the corresponding author/s.

## Author Contributions

EF and MM prepared the manuscript. CP and MK provided substantial editing and conceptual input. All authors contributed to the article and approved the submitted version.

## Conflict of Interest

The authors declare that the research was conducted in the absence of any commercial or financial relationships that could be construed as a potential conflict of interest.
